# An Improved Model Predictive Current Controller of Switched Reluctance Machines Using Time-Multiplexed Current Sensor

**DOI:** 10.3390/s17051146

**Published:** 2017-05-17

**Authors:** Bingchu Li, Xiao Ling, Yixiang Huang, Liang Gong, Chengliang Liu

**Affiliations:** School of Mechanical Engineering, Shanghai JiaoTong University, 800 Dongchuan Road, Minhang Qu, Shanghai 200240, China; bcli@sjtu.edu.cn (B.L.); lingxiao@sjtu.edu.cn (X.L.); huang.yixiang@sjtu.edu.cn (Y.H.); gongliang_mi@sjtu.edu.cn (L.G.)

**Keywords:** model predictive control, current controller, fixed switching frequency, multiplexed current sensor, switched reluctance machine

## Abstract

This paper presents a fixed-switching-frequency model predictive current controller using multiplexed current sensor for switched reluctance machine (SRM) drives. The converter was modified to distinguish currents from simultaneously excited phases during the sampling period. The only current sensor installed in the converter was time division multiplexing for phase current sampling. During the commutation stage, the control steps of adjacent phases were shifted so that sampling time was staggered. The maximum and minimum duty ratio of pulse width modulation (PWM) was limited to keep enough sampling time for analog-to-digital (A/D) conversion. Current sensor multiplexing was realized without complex adjustment of either driver circuit nor control algorithms, while it helps to reduce the cost and errors introduced in current sampling due to inconsistency between sensors. The proposed controller is validated by both simulation and experimental results with a 1.5 kW three-phase 12/8 SRM. Satisfied current sampling is received with little difference compared with independent phase current sensors for each phase. The proposed controller tracks the reference current profile as accurately as the model predictive current controller with independent phase current sensors, while having minor tracking errors compared with a hysteresis current controller.

## 1. Introduction

Due to advantages such as simple and rugged motor construction, high working temperature, and low cost, switched reluctance machine (SRM) drives are becoming viable candidates for industry applications [1,2]. The primary disadvantages of SRMs are torque ripple and acoustic noise. Torque ripple is a consequence of the nonlinear nature of the torque production mechanism [3], while the origin of vibration is the sudden change of radial force when power switches acts [4].

Current profiling is a frequently used method to minimize the torque ripple of SRMs. The current profiles can either be determined through the torque waveform of each phase according to torque-sharing functions [5,6], or derive from a multiobjective optimization considering torque ripple, power loss, and speed range [7]. A high-performance current regulator is then needed to follow the reference profile accurately; the current controller has a relatively high effect on the performance of SRM drive. However, the highly nonlinear electromagnetic characteristics of SRM result from doubly salient structure and magnetic saturation, leading to difficulty in modeling and control [8]. There are two primary approaches to current control in SRMs: hysteresis control [9,10] and pulse width modulation (PWM) control [11,12]. A hysteresis controller is also called bang-bang controller, which tries to keep the phase current within the hysteresis band. Hysteresis controllers are effective in regulating the phase currents with a good dynamic response, but may lead to high current ripples in the low inductance region of SRM due to rapid current rise; moreover, the inverter switching frequency varies unpredictably, which may deteriorate vibration. PWM current controllers have the advantage of fixed switching frequency, and various methods have been used to determine the duty ratio of PWM. In [12], the Proportion-integral (PI) method based on linearized inductance profile was used, and the PI controller had poor performance in face of rapidly changing phase inductance. In [13], a novel high-performance current controller based on iterative learning was proposed without the need of an accurate model. In [14], a sliding mode controller was presented to ensure stability considering modeling errors. Model predictive control (MPC) is a promising method for handling the nonlinear characteristic of SRM, while providing good dynamic, reduced current ripple, and maintaining a fixed switching frequency. In [15], a special predictive control which tried to eliminate the tracking error in a single step was proposed. In [16], a predictive control method adapted to model errors was proposed. In MPC, each phase of SRM can be controlled asynchronously, which enabled MPC for current sensor multiplexing between phases. Sensor umultiplexing leads to a reduced number of current sensors, which not only reduces the cost and volume, but also reduces the additional errors introduced in current sampling due to baseline drift and different magnification factors between sensors.

Current sensor multiplexing technologies have been used in drive systems for permanent magnet synchronous motors, brushless dc motors, and induction motors [17,18,19]. Most sensor multiplexing methods are based on Kirchhoff’s current law (KCL) which states that the sum of currents from all phases is zero, however, it is inapplicable for SRM due to separate windings. For SRM, pulse injection is an effective method for current sensor multiplexing. In [20], a novel phase current reconstruction method was proposed: high-frequency pulses produced by a controller are injected to the switches during the two-phase excitation region to distinguish phase current from bus current; however, the high-frequency pulse injection needs an additional algorithm, and yield increased switch losses and vibration.

This paper focuses on implementing accurate current control under an MPC scheme with reduced current sensors through sensor multiplexing. The proposed method utilizes the feature of independent phase switching control in MPC to realize sensor multiplexing in an alternate sampling manner. Current sensor multiplexing is achieved without additional calculation in MPC scheme. In the two-phase excitation region, the two excited phases have same control frequency but with a skewing of half period; analog-to-digital (A/D) conversions are triggered by period interrupts of PWM counters under continuous-increase-decrease-counting mode, the duty ratio of PWM unions are limited to ensure that one phase is turned off when another phase started A/D conversion. The simulation and experiments are carried out to confirm the feasibility of the proposed method.The contribution of the manuscripts is the realization of current sensor multiplexing in MPC scheme, individual phase is controlled using single-step predictive model, the predictive control steps of adjacent phases are staggered. Current sensor multiplexing is integrated in predictive control without additional control algorithm. The proposed method performs as well as MPC with independent sensors, while achieves lower costs.

## 2. Control System Using a Multiplexed Current Sensor

The current sensor multiplexing is realized with minor adjustment of the SRM drive. The topology of the drive is changed so that no freewheeling and discharging current flowed through current sensor, and the sampling time of each phase was staggered. This section describes the implementation of model predictive current control with only one current sensor in the drive.

### 2.1. Principle of Current Sensor Multiplexing

**Figure 1:**
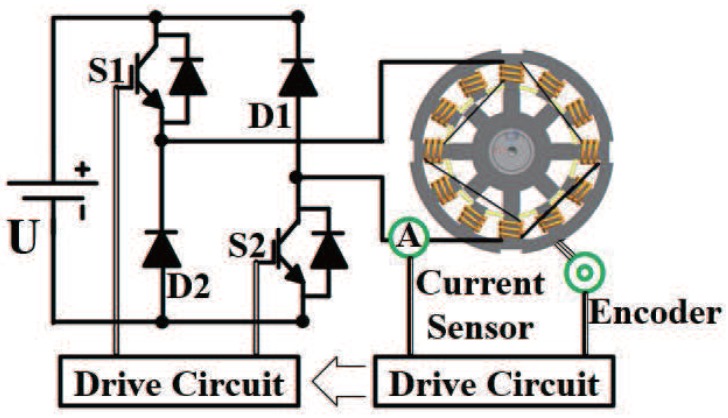
Diagram of a control system with one phase.

The operation fundamental of SRMs is different from other types of motors, such as permanent magnet motors, brushless DC motors, and induction motors. For SRM drive, each phase is independently controlled. Thus, asymmetrical bridge topology is commonly used in SRM converters [21]. Figure 1 shows a conventional 12/8 pole three-phase SRM drive with an asymmetrical half-bridge converter.

**Figure 2:**
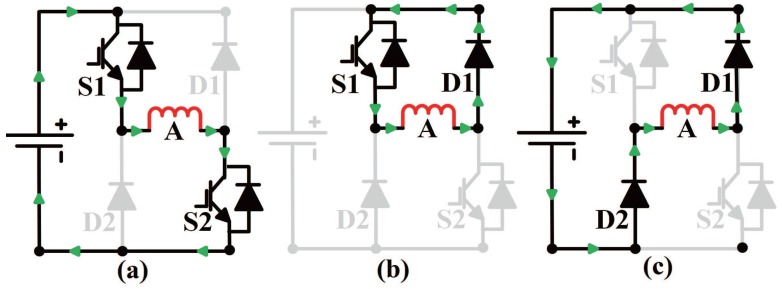
Current flow path of phase A in asymmetric half-bridge converter: (**a**) Mode I: charging; (**b**) Mode II: freewheeling; (**c**) Mode III: discharging.

The phase branch is controlled independently by two switches and operates under three modes, which are named charging, freewheeling, and discharging, represented as mode I, mode II, and mode III, respectively. An abbreviated current path of phase A under each mode is shown in Figure 2.

In mode I, S${}_{1}$ and S${}_{2}$ are both turned on, the current flows from S1 to S2 with positive voltage, and the energy is supplied to phase A; in mode II, S${}_{2}$ turns off while S${}_{1}$ remains on, and the current is cycled with zero voltage; in mode III, S${}_{1}$ and S${}_{2}$ are both turned off, the current flows from D2 to D1 with negative voltage, and the energy flows back to the power supply. If one phase was in mode III and the current decay to zero, it was treated as non-excited. Current regulation was implemented with state transition between three modes. The relation between phase mode and the state of corresponding switches is illustrated in Table 1.

**Figure 3:**
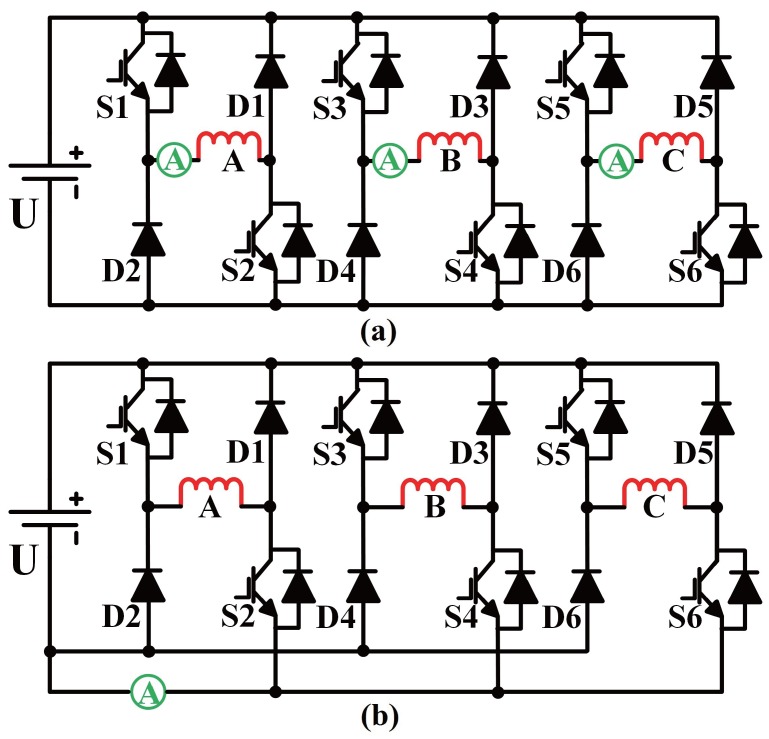
Conventional and modified asymmetric half-bridge converter for three-phase switched reluctance machine (SRM). (**a**) Conventional topology with independent sensors for each phase; (**b**) Modified topology with multiplexed sensor.

In conventional converters, each phase is configured with one current sensor. For the purpose of current sensor multiplexing, the topology is modified to distinguish freewheeling and discharging current from exciting current during the sampling period, as discussed in [20]. The installation of current sensors in conventional topology and modified topology is shown in Figure 3.

**Figure 4:**
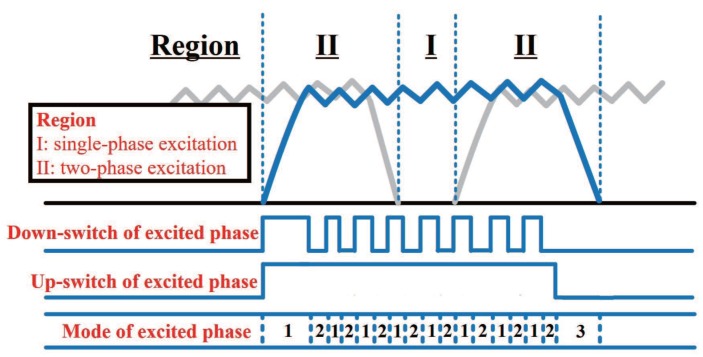
Operation states of SRM in continuous rotation.

In the modified converter, all emitters of the down switches are connected to the negative DC bus through a current sensor. The phases of SRM are excited alternately to carry out continuous rotation; generally, no more than two phases conduct simultaneously. With this configuration, sampling current passes through the bus sensor belongs to one phase if only one phase was excited, while it is a combination of two phases in the commutation stage. The operation of SRM can be categorized into two stages: single-phase excitation and simultaneously two-phase excitation, as illustrated in Figure 4.

In the two-phase excitation stage, the contents of the sampling current depend on the states of both phases. Either the outgoing phase or the incoming phase are in mode II or mode III, and the sampling currents of the sensor are all derived from another phase. All possible combinations of sampling current when phase A and phase B are simultaneously excited and corresponding phase states are summarized in Table 2. The relation between sampling current and phase states can be extended to the commutation stage.

In single-phase excitation stage, the sampling current of the sensor is equal to phase current of the single excited phase when down-switch is on, thus, the result can be used directly in control algorithms. In two-phase excitation stage, the sampling current of the sensor may be the sum of phase currents in two adjacent phases. However, in digital current control, the phase current is sampled with certain frequency instead of continuous sampling, and one can switch one phase to mode II shortly to sampling another phase; that is the basic principle of multiplexing current sensor between two phases. High-frequency pulse injection [20] was developed based on this principle. However, the injected pulse introduced additional current harmonics and deteriorated the vibration and acoustic noise of SRM. With predictive control, the toggle to mode II for the purpose of sensor multiplexing can be integrated in the control scheme in fixed frequency.

### 2.2. Sensor Multiplexing Integrated in Model Predictive Control

#### 2.2.1. Scheme of Predictive Control

Model predictive control (MPC) predicts the future phase current in the next several control steps according to the discrete model of SRM, with average phase voltage as the input. Then, it makes use of predictive behavior to evaluate the predefined cost function, and finally selects the optimal average voltage. The average voltage is implemented through the adjusted duty ratio of PWM union in each control step. The phase voltage equation of an SRM can be given as

(1) $$\;U=R\cdot i+\frac{d\varphi (\theta ,i)}{dt}$$

where *U* is the phase voltage applied on the phase winding, *i* is the phase current, *R* is the winding resistance, φ=L(i,θ)·i is the flux linkage, and L(i,θ) is the phase inductance at position θ with current *i*. The model in the discrete-time domain was derived as

(2) $$\varphi \left(i_{k+1},\theta_{k+1}^{est}\right)=\varphi \left(i_{k},\theta_{k}\right)+\left(U_{k|k}-R\cdot i_{k}\right)\cdot \Delta T=a_{k}\cdot \varphi \left(i_{k},\theta_{k}\right)+\Delta T\cdot U_{k|k}$$

where $a_{k}=1-\Delta T\cdot R/L_{k}$. $U_{k|k}$ is the phase voltage applied during the (k)th period according to prediction in (k)th time. $\theta_{k+1}^{est}$ is the estimated rotor position at (k+1)th time. $i_{k+1}$ and $\varphi (i_{k+1},\theta_{k+1}^{est})$ stand for the current and flux linkage at (k+1)th time, respectively. $i_{k}$, $\theta_{k}$ and $\varphi (i_{k},\theta_{k})$ represent the current, rotor position, and flux linkage at (k)th time, respectively. ΔT is the time period, which was constant in fixed frequency switching.

The predictive model for multiple steps is given in matrix form as

(3) $$\left[\begin{array}{c} \varphi_{k+1}\\ \vdots \\ \varphi_{k+n}\\ \vdots \\ \varphi_{k+h} \end{array}\right]=\left[\begin{array}{c} a_{k}\\ \vdots \\ \prod_{i=0}^{n-1}a_{k+i}\\ \vdots \\ \prod_{i=0}^{h-1}a_{k+i} \end{array}\right]\varphi_{k}+\left[\begin{array}{ccccc} \Delta T & \cdots & 0 & \cdots & 0\\ \vdots & \ddots & \vdots & \ddots & \vdots \\ \Delta T\prod_{i=1}^{n-1}a_{k+i} & \cdots & \Delta T & \cdots & 0\\ \vdots & \ddots & \vdots & \ddots & \vdots \\ \Delta T\prod_{i=1}^{h-1}a_{k+i} & \cdots & \Delta T\prod_{i=n}^{h-1}a_{k+i} & \cdots & \Delta T \end{array}\right]U_{k}$$

where $U_{k}={\left[U_{k|k},U_{k+1|k},\cdots ,U_{k+h-1|k}\right]}^{T}$ is the average input voltage for the next *h* periods, and *h* is the predictive horizon.

Generally, a current reference is predetermined to optimize performance criteria (e.g., torque ripple, copper loss, and torque per current). To minimize torque ripple, a convenient solution for SRMs in instantaneous-torque-control mode is to coordinate the torque production of the individual phases so that the total torque tracks the reference value [3], namely torque-sharing method. The instantaneous torque of individual phase is defined through the suitable torque-sharing function (TSF), then the reference current profile of individual phase is derived according to electromagnetic characteristic. However, in average-torque-control-mode, the elimination of torque ripple depends on conduction angle (turn on/off angle) and reference current, the reference current profile is constant in one conduction period, the elimination method in average-torque-control-mode is less effective but simple than instantaneous-torque-control mode. The elimination of torque ripple may conflicts with other performance criteria, thus, a secondary objective besides torque ripple minimization is specified for collaborative optimization. A commonly used secondary objective is copper loss, obvious advantages in aspects of copper loss and torque ripple were achieved as reported in [4,22]. In this paper, average-torque-control-mode is adopted, the conduction angle and reference current is optimized to reduce torque ripple and copper loss using the method proposed in [22]. The optimal conduction angle and reference current for myriad velocity and average torque are generated offline, and stored in look-up table in matrix form with velocity and average torque as the index. The input voltage needed for SRM to track the predetermined current reference is calculated with a multiple-step predictive model, and the compromise between variance and ripple could be carefully made for better performance, as discussed in [16]. However, the calculation burden is high compared with a single-step model. This paper aims to track reference current rapidly and discuss the feasibility of current sensor multiplexing; thus, a single-step model is used in this paper. The required voltage and duty ratio can be derived from Equation (2).

(4) $$\;U_{k+1|k}=R\cdot i_{k}+\frac{\varphi \left(i_{k+1}^{ref},\theta_{k+1}^{est}\right)-\varphi \left(i_{k},\theta_{k}\right)}{\Delta T},\left(0\leq U_{k+1|k}\leq V_{bus}\right)$$

(5) $$\;D_{k+1}=U_{k+1|k}/V_{bus}$$

where $U_{k+1|k}$ is the average input voltage to reach reference current at (k+1)th period based on (k)th predictive step, $i_{k+1}^{ref}$ is the current reference value in period (k+1)th, $V_{bus}$ is the bus voltage of the inverter, $D_{k+1}$ is the duty ratio for the (k+1)th period. The performance of the predictive model depends on the accuracy of the estimated position. A position filtering is required to eliminate position estimation errors. A digital low-pass filter has been developed and is given by (6) and (7). A moving average of the rotor velocity is calculated from (6), and then the estimated position is obtained from (7).

(6) $$\omega_{avg}=\frac{1}{n}\cdot \sum_{i=0}^{n-1}\frac{\theta_{k-i}-\theta_{k-m-i}}{m\cdot \Delta T}$$

(7) $$\theta_{k+1}^{est}=\theta_{k}+\omega_{avg}\cdot \Delta T$$

**Figure 5:**
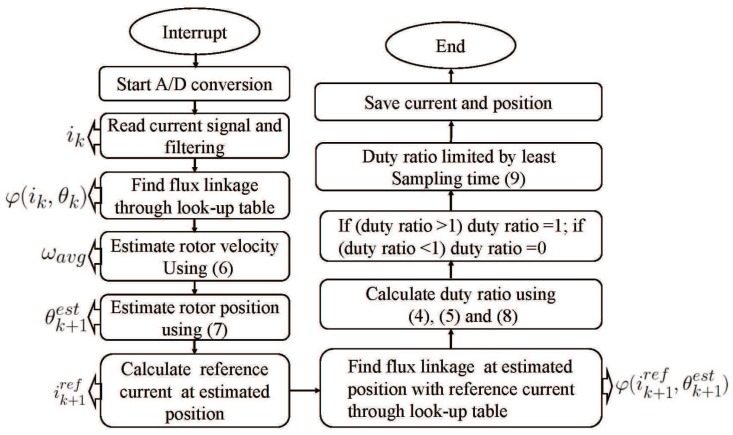
Flowchart of the predictive control.

$\omega_{avg}$ is the estimated rotor velocity using moving average method, m is the interval for single speed calculation, n is the sample size for average speed calculation. The reference current at the estimated position can be derived from predefined current profiles stored in the look-up table. The conduction angle and reference current is extracted from the look-up table according to required velocity and average torque. If the required velocity and average torque are stored in the look-up table, it can be extracted directly. If not, the closet four data points (indexed by two closet velocity and torque) are extracted, then linear interpolations are used to get optimal conduction angle and reference current at given velocity and torque. In current control loop, the reference current is equal to optimal reference current when rotor position is within the conduction interval, while the reference current is zero when rotor position is out of the conduction interval. From the look-up table of electromagnetic characteristic of SRM (φ−θ−i), the flux-linkage at the estimated position under reference current is found. The flowchart of model predictive control is shown in Figure 5.

#### 2.2.2. Model Predictive Current Control with Single Current Sensor

**Figure 6:**
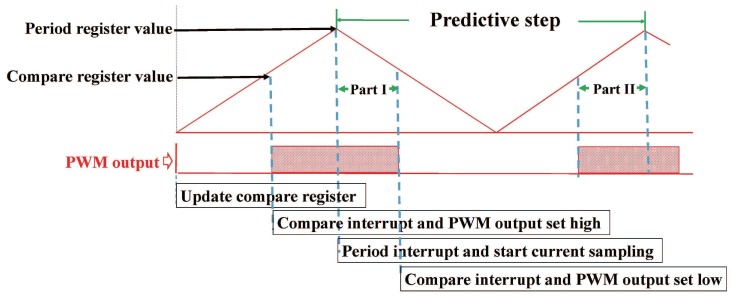
PWM actions and interrupt events triggered by PWM counter.

In the modified converter, the up-switches of excited phase remain open, and the states of down-switches are controlled by the PWM (Pulse width modulation) signal. The PWM union works in continuous-up-down mode, the duty ratios are updated when the PWM counters decrease to zero. The predictive control algorithm executes within period interrupt. The period register value $P^{PWM}$ is decided by time period ΔT and frequency of counter clock. The PWM actions and interrupt events triggered by PWM counter in one PWM cycle are shown in Figure 6. The current sampling is triggered by period interrupt, which is located at the middle of one PWM cycle. The predictive step starts with current sampling and has same frequency with PWM cycle. The predictive step stretches across two PWM periods. The duty ratio in one predictive step is composed of two parts, Part I located at bottom half of the previous PWM cycle and Part II located at first half of the latter PWM cycle.

In the single-phase excitation region, the current flow through the bus sensor equals to the current of the excited phase if down-switch of the excited phase is open, while no current pass through the bus sensor when down-switch of the excited phase is turned off. The sampling current can be used directly in control loops; however, the sampling time should be selected skillfully. In the proposed method, the sampling time is arranged in the middle of a time period. The benefits of this arrangement are:

1. Avoid errors due to current ringings. Current ringings are generated at a switching instant due to parasitics and nonidealities of the switching devices and the inverter. These ringings introduce errors in the sampled current. Current sampling at the middle of a time period leaves enough time for current ringing to decay.
2. Leave enough time for calculating duty ratio using predictive model before compare register updates.

**Figure 7:**
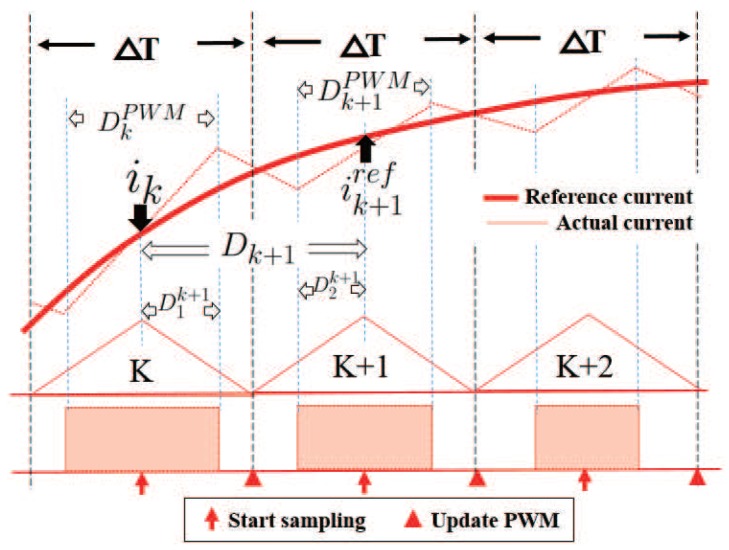
PWM implementation in single phase excited region.

Figure 7 illustrates PWM implementation in single phase excitation region. The (k+1)th predictive step starts with current sampling, and the current sampling located at the middle of (k)th PWM cycle, the duty ratio for (k+1)th predictive step $D^{k+1}$ is derived from (5) to receive reference current value $i_{k+1}^{ref}$ after (k+1)th predictive step. As (k+1)th predictive step stretches across (k)th and (k+1)th PWM periods, $D_{k+1}$ is composed of $D_{1}^{k+1}$ located at bottom half of the (k)th PWM cycle and $D_{2}^{k+1}$ located at first half of the (k+1)th PWM cycle. In Figure 7, $D_{k}^{PWM}$ stand for compare register value at (k)th PWM cycle. To avoid the down-switch of excited phase shutting off during sampling, the minimum duty ratio is set according to the minimum sampling time needed. The (k)th predictive period stretched across two PWM periods: (k)th PWM period and (k+1)th PWM period, the duty ratio of (k)th predictive period is composed of two parts: ${\left(D\right)}_{1}^{k+1}$ located at bottom half of the (k)th PWM cycle and ${\left(D\right)}_{2}^{k+1}$ located at first half of the (k+1)th PWM cycle. The compare register value for (k+1)th PWM is given as

(8) $$D_{k+1}^{PWM}=2\cdot P^{PWM}\cdot \left(1-D_{k+1}\right)+D_{k}^{PWM}$$

**Figure 8:**
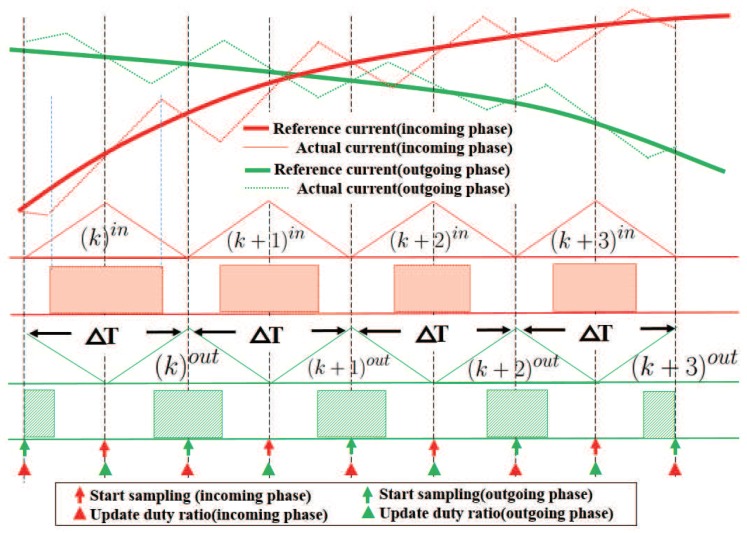
PWM implementation in the two-phases excited region.

$D_{k}^{PWM}$ is compare register value during (k)th period. In two-phase excitation region, the excited phases are either in mode *I* or mode II, the contents of current flow through bus sensor depend on states of two excited phases, as shown in Table 2. The sampling current belongs to one phase if another phase is in mode II, which means the down-switch of another phase was turned off. In the proposed method, the PWM counter of incoming phase is shifted half period from the outgoing phase, the PWM implementation in two-phase excitation region is shown in Figure 8.

When the outgoing phase starts sampling, the incoming phase has just finished the previous period. If the duty ratio of the incoming phase is less than 100%, the down-switch of the incoming phase is turned off when the outgoing phase starts sampling, the sampling current at this moment identifies the phase current of the outgoing phase, which can be used directly in control loops. It is the same case when the incoming phase starts sampling. If we keep the duty ratio of both phases less than 100%, the multiplexing of the current sensor is realized. If the original duty ratio calculated from (5) equals 100% or 0%, the limit of the duty ratio achieves similar form as the pulse injection method; however, in most cases where the original duty ratio generated is between 0% and 100%, the limit of the duty ratio has no effect. The compare register’s value is calculated using (8). The maximum duty ratio plus minimum duty ratio equals one, and the modified register value considering duty ratio limit is given as:

(9) $${\left(D_{k+1}^{PWM}\right)}_{mod}=\left\{\begin{array}{cc} D_{k+1}^{PWM} & \left(D_{samp}\leq D_{k+1}^{PWM}\leq P^{PWM}-D_{samp}\right)\\ P^{PWM}-D_{samp} & \left(D_{k+1}^{PWM}>P^{PWM}-D_{samp}\right)\\ D_{samp} & \left(D_{k+1}^{PWM}<D_{samp}\right) \end{array}\right.$$

(10) $$D_{samp}=t_{samp}\cdot \frac{P_{PWM}}{\Delta T}$$

where ${\left(D_{k+1}^{PWM}\right)}_{mod}$ is modified compare register value to leave enough sampling time during (k+1)th period, $t_{samp}$ is the least time that ensures that the current can be sampled with high precision. $t_{samp}$ depended on the response time of the current sensor and the capability of the A/D converter, $D_{samp}$ is the corresponding number of clock cycles for $t_{samp}$. Generally, $t_{samp}$ was minor compared with period time, and the influence on current control could be neglected.

The current sampling using multiplexed current sensor is integrated into predictive current control. The predictive current control model for single step is given in Equation (4), the parameters in (4) is acquired one by one in the control loop. The steps of predictive current control and parameters gathered in each step is shown in Figure 5. The integration of sampling signal and the predictive model reflected in two aspects: (1) the sampling process is the first step of predictive current control, the sampling signal is then used directly as one of the parameters needed in predictive model, there is no need to change the flowchart of predictive current control; (2) the effective sampling relies on the setups of predictive current control in two-phases excitation region. The control steps of adjacent phases were shifted and duty ratio is limited to leave enough sampling time in predictive current control, such setups ensure that the sampling signal contains current from one phase and can be used directly for the following calculation.

## 3. Simulation and Experimental Results

The feasibility of the proposed current control method is evaluated in simulation and experimental platform. The current sampling result with multiplexed sensor is evaluated. Current waveforms in three control methods—MPC with independent sensors, MPC with multiplexed sensors, and traditional hysteresis control—are compared. A 1.5 kW 12/8 pole SRM was employed in the simulation and experiment, and mechanical parameters of the studied SRM are summarized in Table 3.

**Figure 9:**
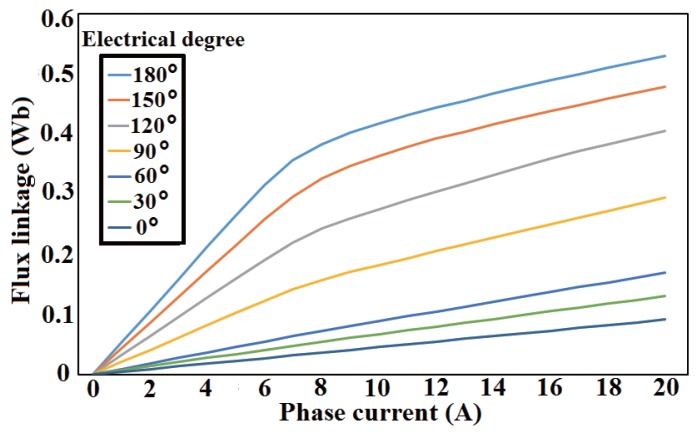
Electromagnetic characteristics of 1.5 kW SRM.

The flux linkage and torque data of the motor obtained using Maxwell software are used, and the electromagnetic characteristic of the studied SRM is shown in Figure 9.

### 3.1. Simulation

**Figure 10:**
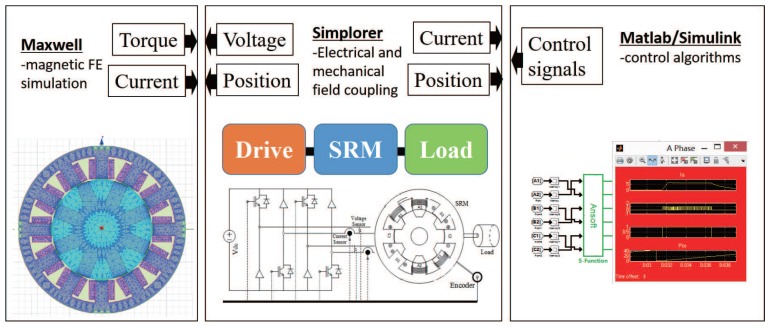
Diagram of multiphysics co-simulation platform for SRM.

To ensure that the simulation is precise, a co-simulation platform, using Matlab, Simplorer and Maxwell, was employed. The 2D finite element model of SRM is built in Maxwell, the power converter and mechanical load is built using multiple-domain simulation software Simplorer, and the control algorithm was executed in Matlab/Simulink to provide the switch signals of the converter. Real-time interaction was undertaken between all software packages. A diagram of the simulation platform is shown in Figure 10. The reference current was set to 6.5 A under a speed of 500 r/min. The turn-on and turn-off angles waere $15^{\circ }$ and $155^{\circ }$ (electrical), respectively. The PWM frequency was set to 10 kHz with sampling time of 1 μs.

**Figure 11:**
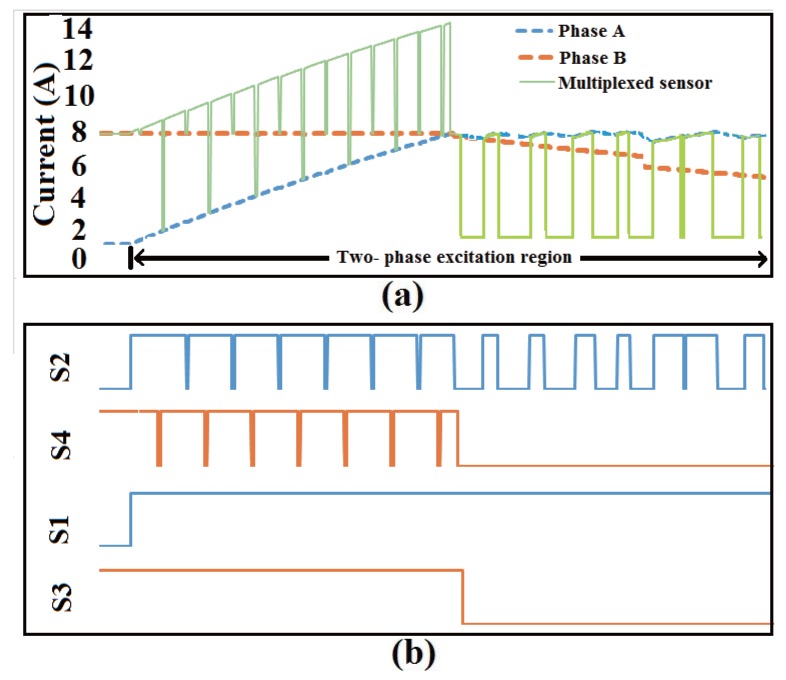
Current sampling process with multiplexed current sensor in two phase excitation region. (**a**) Current waveforms of phase A, phase B and multiplexed sensor; (**b**) Switching states of phase A and phase B.

The current sampling process with multiplexed sensor in two-phase excitation region is shown in Figure 11. The switch states and corresponding sampling current are illustrated. In the two-phase excitation region, two control signals with $180^{\circ }$ phase displacement are generated, which make the down-switches of phases A and B shut off alternately. The current of the bus sensor is equal to one phase current when the down-switch of another phase is shut off. Due to the limit of the duty ratio, the down-switch of the adjacent phase is shut off when the A/D converter started sampling; thus, sampling of two phases are achieved with one sensor. In the one-phase excitation region, the sampling and control of the excited phase is the same as the two-phase excitation region.

**Figure 12:**
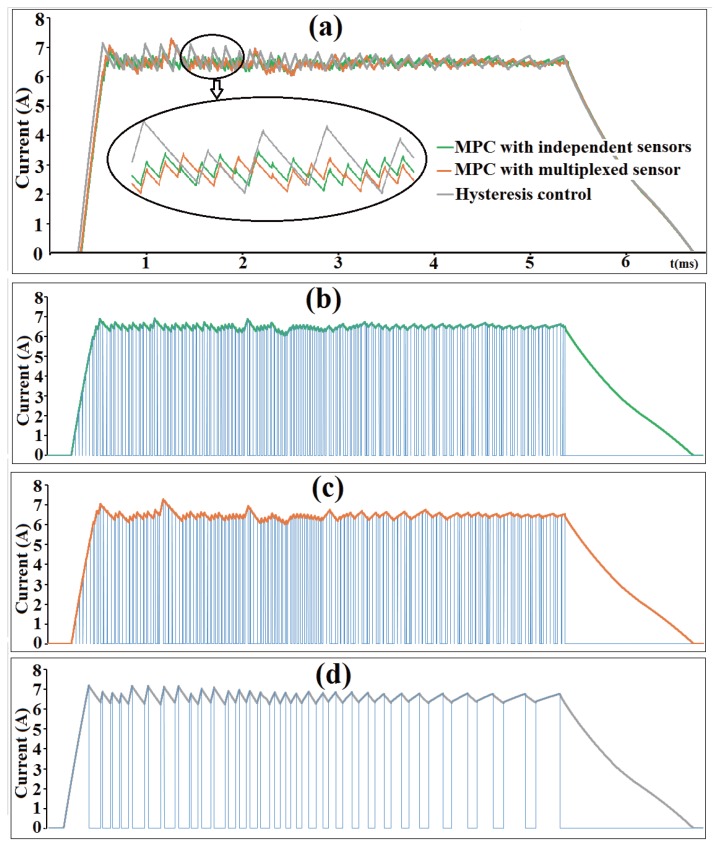
Comparison of three control methods. (**a**) Comparison of current waveform using three methods; (**b**) Action of down switch using model predictive control (MPC) with independent sensors; (**c**) Action of down switch using MPC with multiplexed sensor; (**d**) Action of down switch using hysteresis control.

The performances of current control using three methods are compared, as shown in Figure 12. The three methods are hysteresis control, predictive current control with independent sensors, and predictive current control with multiplexed sensor. Figure 12a illustrates current waveforms using the three methods. The proposed predictive control using a multiplexed sensor has nearly the same performance with that using independent sensors, which implies the feasibility of current sensor multiplexing integrated in MPC. MPC has better performance in tracking the reference current than hysteresis control, especially in the low inductance area. Figure 12b–d illustrates the action of a down-switch using three control methods, respectively. The action frequency of switches in MPC is higher than in hysteresis control, the switch frequency of MPC in the single-phase excitation region is 10 KHz, while the frequency doubled in the two-phase excitation region. However, the switch frequency in hysteresis control is uncertain due to varied phase inductance.

### 3.2. Experiment

A 1.5 kW SRM prototype with the same geometric parameters as the simulation model is employed to build the experimental system, and various experimental results are provided to evaluate the effectiveness of the proposed method. The proposed control method is achieved in Digital Signal Processor (DSP) TMS320F2812 from Texas Instruments (Dallas, TX, USA), which work at a frequency of 150 MHZ. A modified asymmetric bridge inverter is used; however, both independent phase current sensors and a multiplexed bus current sensor are deployed simultaneously in the converter. The bus current sensor is sampled by the built-in A/D converters of the DSP and used directly in the control algorithm. The current data are also saved in the in-board ROM for further verification. The independent phase current sensors are sampled through a data acquisition union (DAQ) from National Instruments. The dc bus voltage was set to 200 V. A 1.5 kW DC motor is used as the mechanical load. The rotor position is obtained by using an incremental encoder with resolution of 1024.

**Figure 13:**
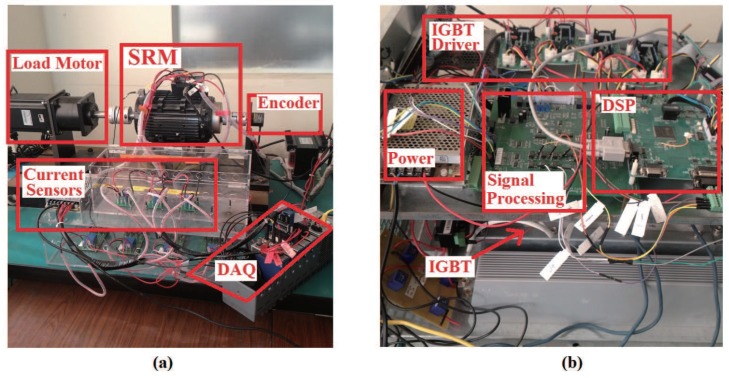
Configuration of the experimental platform. (**a**) Platform structure; (**b**) Driver and controller. DAQ: data acquisition union; IGBT: insulated gate bipolar transistor.

The practical laboratory setup of the experimental system is shown in Figure 13. In the experiments, the executing frequency of the control algorithm is set to 5 kHz considering switching frequency limit of IGBT, and the duty ratio is limited from 5% to 95%. The current reference is set to 6.5 A, and the turn-on and turn-off angles are set to 15${}^{\circ }$ and 155${}^{\circ }$ (electrical degree), respectively.

**Figure 14:**
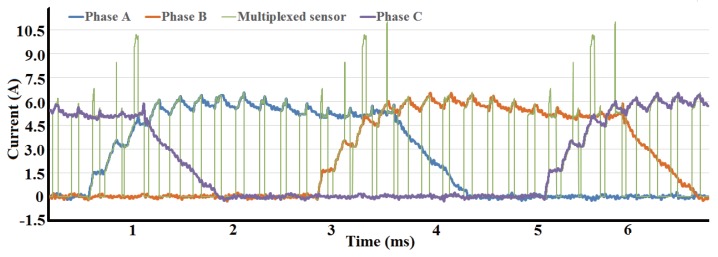
Current waveforms of different sensors.

Figure 14 shows current waveforms sampled simultaneously using independent phase current sensors and multiplexed bus current sensor. The current in multiplexed sensor contained current from one phase when another phase is in mode II or mode III. If both Phase A and Phase B are in mode II or mode III, there is no current passed through multiplexed current sensor, which result in zero value of cyan line. In contrary, if both Phase A and Phase B are in mode I, the current of multiplexed current sensor is the sum of two phases. The control steps of adjacent phases are shifted and duty ratio was limited, which ensure that the current in multiplexed current sensor equals to single phase current. The current waveforms have been filtered to clearly show the relation between current signals from independent sensors and from multiplexed sensor; good consistency is achieved, demonstrating that the single current sensor can be multiplexed in the control system instead of multiple sensors.

**Figure 15:**
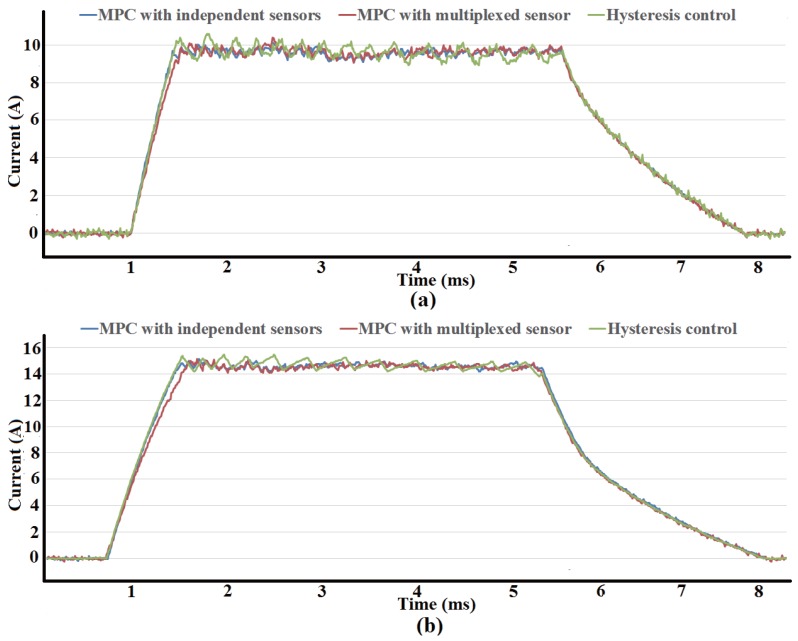
Comparison of current waveform using three control methods at 500 r/min. (**a**) Reference current 10 A; (**b**) Reference current 15 A.

**Figure 16:**
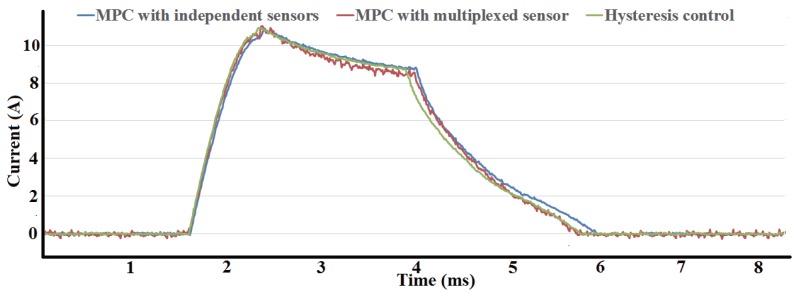
Comparison of current waveform using three control methods at 1000 r/min wit reference current 10 A.

The performance of three control methods, MPC with independent current sensors, MPC with multiplexed current sensor and hysteresis control with independent current sensors, are compared in different condition. Figure 15a shows the current waveforms under three control methods with reference of 10 A at 500 r/min. The performance with reference of 15A, a more saturated condition, at same speed is shown in Figure 15b. In both condition, MPC has better performance than hysteresis control in terms of tracking errors. After reference current is arrived, MPC with multiplexed current sensor have similar results as MPC with independent sensors, which confirm the validity of the proposed current sensor multiplexing method. However, before reference current is arrived, current ripples occurr in MPC with multiplexed sensor as a result of duty ratio limits. The duty ratio is limited to leave sampling time for another simultaneously excited phase in MPC with multiplexed sensor, while the duty ratio in MPC with independent sensors is 100The current waveforms under three control methods with reference of 10 A at 1000 r/min is shown in Figure 16. MPC with independent sensors has same result as hysteresis control, however, phase current when using MPC with multiplexed sensor is smaller than other methods. The duty ratio tends to 100% as speed increase, the effect of duty ratio limit in MPC with multiplexed sensor becomes obvious.

## 4. Conclusions

A current control method of SRM using MPC scheme with sensor multiplexing method was proposed. The proposed method exploits the flexibility of MPC in sampling time. The principle of current sensor multiplexing was introduced. The implementation of MPC with a multiplexed sensor was detailed, and the topology of a converter was modified to distinguish currents from simultaneously excited phases. MPC was executed in parallel in adjacent phases; however, the control steps of the two phases were staggered. The duty ratio of PWM was limited to make enough time for sampling. Both simulation and experiment were done to validate the effectiveness of the proposed method. The results showed that sampling current from a multiplexed current sensor was coincident with that from independent phase current sensors. MPC using a multiplexed current sensor has similar performance as using independent phase current sensors in phase current control. The switching frequency is fixed when using MPC. In simulation and experimental tests, both MPC with independent sensors and MPC with multiplexed sensor have minor current ripple than hysteresis current control in tracking reference current. MPC using a multiplexed current sensor was an improved method to reduce the cost with guaranteed performance.

## Figures and Tables

**Table 1:** Relation between current mode and switch states of Phase A.

Phase A	S${}_{1}$	S${}_{2}$
Mode I	ON	ON
Mode II	ON	OFF
Mode III	OFF	OFF

**Table 2:** Contents of sampling current and phase state.

Combination	Sampling Current	Phase A	Phase B	Phase C
1	$i_{a}$ + $i_{b}$	Mode I	Mode I	Not excited
2	$i_{b}$	Mode II or Mode III	Mode I	Not excited
3	$i_{a}$	Mode I	Mode II or Mode III	Not excited
4	0	Mode II or Mode III	Mode II or Mode III	Not excited

**Table 3:** Geometric information for the 1.5 kW SRM.

Parameter	Value
Number of rotor poles	8
Number of stator poles	12
Stator outer diameter	130 mm
Rotor outer diameter	77.4 mm
Stator outer diameter	130 mm
Stator pole arc	$15^{\circ }$
Rotor pole arc	$15.9^{\circ }$

## Abbreviations

The following abbreviations are used in this manuscript:

SRM	Switched reluctance machine
A/D	Analog-to-Digital Converter
PWM	Pulse Width Modulation
MPC	Model Predictive Control
KCL	Kirchhoff Current Law
DAQ	Data Acquisition
IGBT	Insulated Gate Bipolar Transistor
